# Notes on Our Visits to Colleges and Hospitals in Philadelphia

**Published:** 1872-08

**Authors:** 


### NOTES ON OUR VISIT TO COLLEGES AND HOSPITALS IN PHILADELPHIA.

[*Continued.*]

We next visited that fine institution, the Jefferson Medical College. It is unnecessary to say anything in regard to the buildings, etc. Passing through the college building, we came to the clinical department, where we found that great and distinguished American surgeon, Prof. Gross, delivering a lecture upon syphilis. Prof. Gross is a charming lecturer, is perfectly at home in every department of surgery, and has a rich, impressive voice and style. It does one good to listen to such a teacher, whose utterances come with all the power of a great medical authority, and whose genial spirit sparkles and glows in every sentence and word.

In the office of the Dean, we had the pleasure of meeting Prof. Rand, with whom we had a delightful social interview. Our impression of Prof. Rand cannot be easily erased. We could see a noble manhood impressed upon every lineament of his highly intellectual face. Sterling integrity, of granite solidity, enthrones itself upon his brow, and beams from his piercing eye.

We were disappointed in not meeting our old friend, Prof. Biddle—a gentleman we have known and loved since our student days. We also failed to see the two young Professors, Meigs and Pancoast, whose fathers were the friends of the writer. We desired to extend the heart's warm greeting to their sons, in honorable memory of the distinguished dead—those noble men who have done so much for humanity and science. It is a pleasure to know that their places are occupied by noble sons, whose talents they transmit to science, and whose names they nobly represent. We were impressed more than ever with the thought—the principle—that high intellectual endowments united with high moral attributes, are the only sure bases of character and tone in a medical—or any other—institution. Stripped of these, they shrivel and decay, shunned by all true men. Trickery, cunning and time-serving policy dooms an institution to inevitable ruin; while high, noble and pure purposes bring a harvest of honor and success, rich in its results to humanity and science.

The Jefferson Medical College, like the University, besides the clinics of the institution proper, has great and peculiar advantages which are afforded by the numerous hospitals and charitable institutions of the city. We here purpose speaking of only a few of these, by way of showing these advantages:

*Pennsylvania Hospital.*—This is the oldest hospital of Philadelphia, and among its first board of managers appears the name of Benjamin Franklin. The hospital covers a square of ground of four acres. The object of its founders was to provide relief for the poor suffering with incurable diseases—those arising from infection, or syphilis, &c., being excluded. Tickets for admission are free, and the clinics are held twice a week, being largely attended by the students of the medical colleges. The present medical staff is an excellent one—consisting of four attending physicians, four attending surgeons, and three resident physicians. It has a large library attached. It is said ninety thousand patients have availed themselves of the benefits of this hospital since its establishment—fifty thousand of these having been supported exclusively by its charity.

*The Philadelphia Hospital.*—This institution is situated on the west bank of the Schulkill River, within a few hundred yards of the new buildings now being erected for the University of Pennsylvania. It consists of four buildings five hundred feet long and three stories high. A large farm is also attached, which is cultivated by the inmates. It is believed to be the only hospital in this country in which the plan of continuous services of its attending staff is carried out. It is supported by the city, and is under the charge of a Board of Guardians. It embraces medical, surgical and obstetrical wards, with a children asylum—also an insane department. The medical staff is composed of four physicians, four surgeons, four obstetricians, a pathologist, microscopist, and a resident in charge of the insane department. Each of these gentlemen is continuously on duty. Clinical lectures in medicine, sugery, and the diseases of women and children are delivered on Wednesdays and Saturdays, throughout the year, except in July and August. Different gentlemen of each class also receive ward classes for bed-side instruction. The members of these classes are taken to the bed-side of the patients, instructed to examine and prescribe for them as in their own private practice, while the various methods and instruments of physical explorations, including the clinical use of the microscope, are taught by others. There are also attached to the services of this hospital thirteen resident physicians—young gentlemen who, with a number of chemical clerks, are selected from among the graduates of the regular medical schools of the city, for the term of fifteen months, during which time they pass in succession through the different wards, in which they are thus enabled to profit by the most varied experience. The chemical clerks are chosen by the medical officers whom they assist, and possess opportunities for the study of disease nearly equal to those of the resident physicians. The hospital proper has seven hundred beds connected with it. During the year 1868–1869 5,832 patients were treated—241 women were confined. Out-door relief was given to 85,232 persons.

The arrangements throughout are excellent—making it one of the best institutions for the afflicted in the country, and a most desirable school for a student. It requires $250,000 annually for its support. The hospital staff, for excellence of character and ability, falls short of no other similar institution. Among these gentlemen our readers would recognize the name of that accomplished gentleman and physician, Dr. James Tyson, who for many months has been one of the corresponding editors of the COMPANION.

*The Episcopal Hospital* is located at Front street and Lehigh Avenue; is massive and of handsome appearance. This hospital draws its support from individual charity, and is a proud monument to the Christian benevolence of its founders and supporters. It is under fine management, being conducted by a board of twenty-four Christian gentlemen, at whose head presides the Protestant Episcopal Bishop of the Diocese as the President *ex officio*. The professional staff consists of four attending physicians, four attending surgeons, with eight resident physicians, and an apothecary. The "Hancock Ward" of this hospital was founded by bequest of Miss Grosby, for the treatment of patients with diseases of the chest. This is a general hospital—no distinction of creed or color is made in the admission of patients. During the year 1860 nine hundred and thirty-seven patients were admitted and a large number of out-patients (7,392) prescribed for. The hospital at present has one hundred beds occupied.

*St. Joseph's Hospital* is under the control and direction of the Sisters of Charity, and is supported entirely by voluntary contributions. It has apartments for surgical, medical and obstetrical treatment, and in addition to its public wards, has rooms handsomely fitted for patients who are able to pay, and wish to avail themselves of its great advantages of treatment, etc., etc. Patients of all religious belief here find a cordial welcome and tender attention from these Sisters of Charity, and can, if they desire, choose from any denomination their spiritual advisers, in their affliction, while, "without money and without price," their bodily wants are supplied. The medical staff comprises four physicians and four surgeons, with two obstetricians—an excellent body of medical gentlemen.

*St. Mary's Hospital* is under the supervision and control of the Franciscan Sisters, and sustained entirely by individual contributions. It was founded in 1866, and since that time 1,910 patients have received the benefits of its charity. The management of this hospital is not surpassed, while for ability and zeal the medical staff is not excelled. In its dispensary department, since its inauguration, 8,654 patients have been treated. Its medical staff consists of four attending surgeons and four physicians for the dispensatory department. We are pleased in having obtained the consent of Dr. W. W. Keen, one of their number, as a corresponding editor for the COMPANION. Dr. K. is an accomplished gentleman and an able physician.

*Wills Hospital.*—This hospital is situated on Rose street, between Eighteenth and Nineteenth streets, is finely located, with handsome grounds attached. This institution was founded by the late James Wills—bequeathing to the city a large sum for the purpose. Daily consultations are held for out-door patients, who come in large numbers to avail themselves of this charity. Regular clinics are held two days in each week. The surgical staff comprises some of the ablest surgeons of the city.

*The Children's Hospital.*—This institution is supported entirely by private subscriptions, and has accommodations for over fifty in-door patients, with dispensary for out-door patients. A ladies' visiting committee is a feature of importance. All poor children under twelve years of age are received, save those only who need surgical treatment, or have contagious diseases. The medical staff is composed of nine gentlemen.

*Charity Hospital of Philadelphia.*—This institution is supported by individual donations—patients being required to pay a small amount for board. It is hoped, however, by the trustees, to be able soon to afford urgent cases gratuitous service. Medical advice and medicines are given gratuitously at the daily clinic. It is an excellent institution. The medical boards consist of thirteen of the most accomplished medical gentlemen of Philidelphia.

*Presbyterian Hospital* is situated in West Philadelphia, and at present the hospital has been opened in temporary buildings already found on the grounds. The new building, when completed, will accommodate 400 patients, and will form a hospital not to be excelled by any in the country. The endowment fund has already reached four hundred thousand dollars—three hundred thousand of which was subscribed by one gentleman. The medical staff consists of four physicians, four surgeons, four consulting physicians, two consulting surgeons, two opthalmic surgeons, two obstetricians, and two pathologists. This hospital is open to all classes of people, without regard to creed or color.

*The University Hospital* is being planned, and will be erected on a square of fifteen acres of ground, very near the Philadelphia Hospital. The ground was given by the city to the trustees of this time-honored institution, who propose to erect five or six of the handsomest college buildings perhaps in the world—which will enable the trustees and faculty of the medical department to insure instruction far superior to that now imparted in its old halls on Ninth, between Chestnut and Market streets. Students will not only have the advantages of this hospital, which will number five hundred beds, but of the Philadelphia Hospital, on the adjoining grounds. It is gratifying to the alumni of the University, to see these evidences of progressive energy. As time rushes on, instead of remaining at a stand-still, jostled on all sides by younger and no less active schools, the old University spreads out—keeping up in material progress with the rapid strides in every department of science and successful teaching.

In our next, we may speak of our brief visit to New York.

